# US Consumers’ Awareness, Purchase Intent, and Willingness to Pay for Packaging That Reduces Household Food Waste

**DOI:** 10.3390/foods12234315

**Published:** 2023-11-29

**Authors:** Korey Fennell, Guanqi Lu, Monireh Mahmoudi, Euihark Lee, Eva Almenar

**Affiliations:** 1 School of Packaging, Michigan State University, East Lansing, MI 48824, USA; fennel22@msu.edu (K.F.); mahmou18@msu.edu (M.M.); leeeuiha@msu.edu (E.L.); 2 College of Agriculture and Natural Resources Statistical Consulting Center, Michigan State University, East Lansing, MI 48824, USA; luguanqi@msu.edu

**Keywords:** education, food freshness, packaging design, packaging technologies, population segments

## Abstract

Food waste is a barrier to the development of sustainable food systems, and a large portion of it occurs at the household level. Household food waste can be decreased by using appropriate packaging. Despite the high rate of food waste in US households, little is known about how packaging affects this. This study assessed US consumers’ awareness of how structural packaging designs and technologies affect food freshness and their willingness to purchase and to pay extra for packaging designed to reduce household food waste. To gather data, 1000 US consumers were surveyed online. Responses were analyzed overall and by population segments. The impacts of only 3 out of 15 structural packaging designs on maintaining food freshness were known by >50% of consumers. Regarding packaging technologies, while 78% of consumers knew about the impact of vacuum packaging on maintaining food freshness, just 27.6, 23, and 16% knew how modified atmosphere packaging, active packaging, and aseptic packaging affected food freshness. Only 32% of consumers knew that intelligent packaging provides information on food freshness. Just 9% of consumers recognized that foods in plastic pouches and cans possess the same food freshness. Approximately 91% of consumers will always/sometimes buy food in most of the above packaging technologies after learning about them. Half were willing to pay more for food in packages that reduce household food waste, and 40% may. Differences (*p* ≤ 0.05) and two-way interactions were observed between population segments. This study’s findings can help develop new packaging, education campaigns, and policies to reduce household food waste in the US.

## 1. Introduction

Food waste poses a significant obstacle to the development of sustainable food systems. Solely in the US, 119 billion pounds of food are wasted annually, amounting to a loss of more than USD 408 billion [1]. A significant part of this waste (43%) occurs at the household level [2]. Therefore, household-level interventions can greatly reduce food waste in the US. Packaging is one of these interventions [3,4]. Packaging can help by functioning as a container for preservation, making the contents simple to access, and providing cooking instructions [5]. As a container for preservation, both structural packaging design and packaging technology play a key role in food waste reduction.

Packaging design involves a combination of structural, graphical, and verbal elements [6]. The structural elements, material, and format are the ones that affect food shelf-life and hence have the greatest impact on household food waste. Specific packaging materials and packaging formats are chosen to package food based on the matching of their inherent qualities (e.g., barrier, vacuum resistance) with the intended shelf-life extension (short vs. long) [7,8,9]. Despite the significance of consumer awareness of the role structural packaging design plays in food shelf-life extension, which can greatly aid in making the proper choices when selecting packaging to reduce household food waste, there is little to no information about it. Previous research has focused on investigating the power of structural packaging design in affecting the consumers’ perceived quality [6,10,11], taste [12,13,14], safety [15], and healthiness [6,13] of food. However, these studies have no connection with food shelf-life extension. To the authors’ knowledge, only two studies on consumer behavior have examined the intersection between structural packaging design and food freshness. One reports that South Africans connected the glass jar, carton box, and plastic pouch with long-term food storage because they had observed these packages containing durable food products [15]. The other study found that some Australians think packaging keeps food fresh, while others think it degrades food faster after seeing numerous package styles [16]. Hence, only a few studies with inconclusive findings are currently available in the literature. It is crucial to get to know more about consumers’ awareness of the role structural packaging design plays in food shelf-life extension if strategies that can significantly contribute to reducing household food waste are to be developed.

A variety of packaging technologies are available at the marketplace to ensure food safety and extend food shelf-life. These include vacuum packaging (VP), active packaging (AP), modified atmosphere packaging (MAP), intelligent packaging (IP), aseptic packaging (ASP), and retort packaging (RP) [9,17,18,19,20,21]. Despite availability, consumers’ awareness, acceptance, purchase intent, and willingness to pay are all required for these packaging technologies to significantly reduce household food waste.

Regarding consumers’ awareness, knowledge about the existence of these technologies in the marketplace and their impact on food shelf-life are both essential for household food waste reduction. As shown in Table 1, information on packaging technology recognition is provided by survey studies about VP, MAP, AP, and IP. These studies attest that recognition varies greatly among technologies. While VP is well recognized [10,22,23], recognition rates of 16%, 4–22%, and 17–28% have been reported for MAP, AP, and IP, respectively [24,25,26,27,28]. Regarding ASP and RP, there are no survey studies on consumers’ attitudes towards these two technologies in the literature despite being able to extend the shelf-life of many food products and thereby reducing household food waste. Consumers’ knowledge on the impact of these packaging technologies on food shelf-life extension is unknown, except for VP’s impact (Table 1; [10,22]). Therefore, little is known about how consumers perceive the impact of packaging technology on food shelf-life relative to its recognition, even though this information is crucial to understanding consumers’ ability to choose packaging technologies during their shopping decisions in a way that helps reduce household food waste.

Table 1 shows that consumers’ acceptance of MAP, VP, AP, and IP is well known [22,23,24,25,27,29,30,31,32,33]. The misalignment between the lack of knowledge of the impact of packaging technologies on food freshness and the acceptance of these technologies has resulted from the latter being assessed after consumers were provided with written or verbal information on the packaging technologies right before responding [23,27,31]. However, consumers’ acceptance of a packaging technology does not guarantee its purchase. Hence, knowledge of purchase intent may be more trustworthy than knowledge of acceptance to forecast how each of these packaging technologies will position itself in the market. The purchase intent of AP and IP has been reported. This ranged from 9 to 77% depending on the consumers’ geographical location and, generally, it was higher for IP than AP (Table 1 [24,26,27,34]). However, the purchase intent of VP, MAP, ASP, and RP is still unknown.

**Table 1:** Survey studies on consumer behavior towards packaging technologies that can reduce food waste.

Investigated PKG Technology (PT)	Effect of the PT on Food Freshness	Packaged Food Product	Country Where the Study Took Place	Consumers’ Awareness—Knowledge of PT Presence in the Marketplace	Consumers’ Awareness—Knowledge of PT Impact on Maintaining Food Freshness	Consumers’ Acceptance of PT	Consumers’ Purchase Intent of PT	Consumers’ Willingness to Pay Extra for a PT That Extends Shelf-Life	Consumers’ Demographics	Consumers’ Psychographics	Reference
AP (n = 94)	Shelf-life	Fresh-cut cantaloupe	US	Not investigated	Not investigated	Investigated	Not investigated	Investigated	Investigated	Not investigated	[33]
AP (n = 2325)	Shelf-life	Pita, bagged salad, salmon steaks, cheese, etc.	US	Not investigated	Not investigated	Not investigated	Not investigated	Investigated	Investigated	Not investigated	[31]
AP and IP (n = 372)	Quality and safety	Food in general	Poland	Investigated	Not investigated	Investigated	Investigated	Investigated	Investigated	Not investigated	[24]
AP and IP (n = N/A)	Reduce HFW	Food in general	Italy	Not investigated	Not investigated	Not investigated	Investigated	Not investigated	Not investigated	Not investigated	[34]
AP and IP (n = 814)	Shelf-life	Cheese	Ireland	Investigated	Not investigated	Investigated	Investigated	Investigated	Investigated	Not investigated	[27]
AP and IP (n = 488)	N/A	Food in general	Poland	Investigated	Not investigated	Not investigated	Not investigated	Not investigated	Investigated	Not investigated	[28]
AP and IP (n = 365)	Shelf-life	Food in general	Turkey	Not investigated	Not investigated	Investigated	Not investigated	Investigated	Investigated	Not investigated	[29]
AP and IP (n = 865)	Freshness and quality	Food in general	Latvia	Investigated	Not investigated	Not investigated	Investigated	Investigated	Not investigated	Not investigated	[26]
MAP (n = 106)	Shelf-life and color	Ground beef	US	Investigated	Not investigated	Investigated	Not investigated	Investigated	Not investigated	Not investigated	[25]
MAP (n = 212)	Shelf-life and color	Ground beef	US and Germany	Not investigated	Not investigated	Investigated	Not investigated	Investigated	Not investigated	Not investigated	[30]
VP, MAP, and AP (n = 2520)	Safety	Beef	Five European countries	Not investigated	Not investigated	Investigated	Not investigated	Not investigated	Investigated	Investigated	[23]
VP (n = 108)	Shelf-life	Beef	Canada	Investigated	Investigated	Investigated	Not investigated	Investigated	Investigated	Not investigated	[22]
VP (n = 547)	Quality	Cheese	Lebanon	Not investigated	Investigated	Not investigated	Not investigated	Investigated	Investigated	Not investigated	[10]
VP (n = 93)	Quality	Beef	US	Not investigated	Not investigated	Investigated	Not investigated	Investigated	Investigated	Not investigated	[32]

N/A = not applies.

In addition to having the intention to buy, consumers must be willing to pay more for packaging technologies that reduce food waste, because they come at an additional cost. Due to these technologies’ ability to extend food shelf-life, some research presented in Table 1 has found that consumers are prepared to pay more for MAP [25], AP and IP [24], AP [31,33], and VP [10]. However, other researchers have found the opposite [22,26,27,29]. The respondents’ different geographic regions could be the cause of these contradictory results. Regardless, consumers’ decisions were made based on the claim of shelf-life extension being caused by the packaging technology. Hence, there is no study in the literature that reports on consumers’ willingness to pay extra for food in packaging designed to reduce food waste when they are asked directly about it.

The above shows that further research is needed to determine how packaging can reduce household food waste. Understanding this relationship can positively influence consumers’ packaging selections and thus reduce food waste. Despite the US being among the leaders in food waste, the literature lacks information on consumers’ knowledge and attitudes towards structural packaging designs. As for packaging technologies, US consumers’ knowledge and attitudes towards IP, ASP, and RP is unknown, and little is known about AP, VP, and MAP (Table 1; [25,30,31,32,33]). In addition, differences among segments of the American population for only two of the six technologies are reported, and the divides were based on demographics, so no information regarding psychographics was provided to help decision makers take appropriate actions that might accelerate the reduction of food waste (Table 1). The authors hypothesize that US consumers are not aware of the impact of structural packaging designs and packaging technologies on food freshness, but their learning about it will increase their willingness to buy and pay for food in packaging that reduces food waste. However, this willingness will differ among population segments. The current study aims to reject or accept this null hypothesis and fill in the previously mentioned knowledge gaps by investigating the followings: (1) US consumers’ awareness of the impact of structural packaging designs (material + format) and packaging technologies (IP, AP, MAP, VP, ASP, and RP) on food freshness; (2) US consumers’ purchase intent of the aforementioned packaging technologies; (3) US consumers’ willingness to pay extra for food products in packaging designed to reduce household food waste; and (4) differences in the above caused by population segments. Understanding consumer awareness, purchase intent, and willingness to pay for food-waste-reducing packaging and incorporating these insights can greatly aid in the creation of innovative packaging, the creation of successful educational initiatives, and the formulation of new legislation aimed at reducing food waste in households.

## 2. Materials and Methods

### 2.1. Survey Questionnaire Development

A draft two-part questionnaire and its associated files (screening questions and consent form) were developed and approved by the Michigan State University Institutional Review Board (protocol code STUDY00002025) before being shared with representatives from the Environmental Research and Educational Foundation (EREF), AMERIPEN, and others in the packaging industry for feedback. A pilot test was conducted with approximately 50 people differing in background (education level, gender, race, age, etc.) who met the inclusion requirements of the questionnaire (to be 18 years old or older, to be primary shoppers in their households, to purchase groceries at least once every other week, and not to be a packaging expert or related to one) to gauge additional feedback. The questionnaire was improved accordingly when appropriate.

After responding to the screening criteria questions, participants read a brief introduction that explained the length of the questionnaire, the authors’ definition of household food waste (“Household food waste is food that is fit for consumption but is discarded in the household including meal leftovers but excluding non-edible parts of food (e.g., banana peel, meat bones”), and examples of household food waste states. Participants were additionally instructed to answer the questionnaire questions based on their own viewpoints or personal experiences in their respective households. Finally, participants were encouraged to respond “I don’t know” if they were not aware of a specific interaction between packaging and household food waste.

The first part of the questionnaire consisted of a list of questions of a “choose one guide-type response” to collect participants’ demographic segments, including age, race, education, ethnicity, gender, marital status, income, disability status, household size, and residency (state) as well as participants’ psychographic segments, including grocery shopping frequency, grocery shopping method, and contribution to reducing household food waste along with the reasoning behind it. The second part of the questionnaire consisted of 18 questions, which included a list of ordinal scale and categorical questions (Likert scale and guide-type questions). This part consisted of the four sections described below.

#### 2.1.1. Assessing Consumers’ Awareness of the Impact of Structural Packaging Design (Format + Material) on Food Freshness

Participants were shown photos of three common packages for cherries, milk, bread, chicken, and peanut butter (Table 2) and then asked to compare these using the freshness-related choices “this package can keep my product fresh the least”, “neither the freshest nor the least fresh”, and “this package can keep my product fresh the most”. The selected choice was dropped for the remaining packages. The packages of each food product were shown on a different page of the online questionnaire. Only one page was displayed at a time, and the photos occupied 50% of it. Both the material and format were provided underneath each photo.

#### 2.1.2. Assessing Consumers’ Awareness of the Impact of Packaging Technologies on Food Freshness

Two packages were displayed on each page of the questionnaire. Only one page was displayed at a time, and the photos occupied 60% of it. One of the two packages showed the food product packaged in the packaging technology under assessment (MAP, AP, IP, RP, ASP, and VP) and the other one showed the same food product without the packaging technology (Table 3). Participants were asked to choose between the packaged products presented which one of the two alternatives in a pair keeps the food product fresh for longer. Participants were provided with no information other than left (package A) and right (package B). The possible responses to choose from were: A, B, I don’t know, and No difference.

#### 2.1.3. Assessing Consumers’ Purchase Intent towards Packaging Technologies after Being Educated

The same pair of photos per technology as in Section 2 along with text that explained the difference between the two packages (Table 4) was shown to the participants for educational purposes. Only one page of the questionnaire was displayed at a time, and the photos occupied 50% of it. Then, participants were asked to respond to the question: “How often would you purchase food product A or another food product in technology X when found in the store?” The possible responses to choose from were: always, sometimes, and never.

#### 2.1.4. Assessing Consumers’ Willingness to Pay Extra for Food Products in Packaging Designed to Reduce Household Food Waste

Consumers’ willingness to pay extra for food products in packages designed to help reduce food waste was investigated by asking participant exactly this and giving yes, no, and maybe as possible responses.

### 2.2. Data Collection

Qualtrics was utilized for both the design and implementation of the online survey questionnaire. The questionnaire was launched in November of 2022. Approximately 2700 respondents from across the continental United States who were willing to take surveys in exchange for an economic reward and met the screening criteria received the link to the survey sent by Ugam, as per the agreement with Qualtrics. They were narrowed down to 1000 based on the reliability of their responses. These participants matched the splits from the US census [35] within ±2.0% for gender, age ranges, education levels, annual income, marital status, household size, disability, and region (e.g., state you live in) to have a fair representation of the US population.

### 2.3. Statistical Analysis

Principal Component Analysis (PCA) was performed to examine the data from Section 2.1.1, Section 2.1.2 and Section 2.1.3 for potential correlations between the factors in each of these three question blocks using SPSS (IBM^®^ Statistics 29, Armork, New York, NY, USA). The PCA results for the first two question blocks (Section 2.1.1 and Section 2.1.2) showed that factor correlation was not appropriate, indicating a significant variation in consumers’ knowledge about the effects of packaging technologies and structural designs on food freshness (referred to as the “KNOWLEDGE_TECH” and “KNOWLEDGE_STR” variables) between food products. Based on the findings of the pilot test, which indicated that knowledge depended on the type of food product, the inappropriateness of PCA was anticipated. The Kaiser–Meyer–Olkin estimate for the PCA conducted for question block Section 2.1.3 was 0.753, indicating that the consumer, rather than the food, is primarily responsible for consumers’ willingness to purchase food products in packaging that preserves their freshness for a longer period (referred to as the “PURCHASE INTENT” variable). It was inappropriate to extract Cronbach’s Alpha for “KNOWLEDGE_STR” and “KNOWLEDGE_TECH” because food itself has a significant influence on the consumer’s understanding of how structural packaging designs and packaging technologies affect food freshness. The retrieved Cronbach’s Alpha for “PURCHASE INTENT” was within an acceptable range (0.657), and this was expected due to the dependence of the data on the consumer, rather than the food.

Participant data were analyzed using SAS 9.4 (SAS Institute Inc., Cary, NC, USA). The categorical responses were analyzed using a chi-square test of independence for each individual population segment (i.e., age, gender, education, ethnicity, etc.) with the PROC FREQ procedure. The ordinal responses were analyzed using a chi-square test of independence for each individual population segment (i.e., age, gender, education, ethnicity, etc.) and their two-way interactions using a PROC LOGISTIC procedure. The chi-square test *p*-value < 0.05 indicated a significant association between the response and the population segments. For binary responses, the model used was binary logistic regression, and for other ordinal responses with more than 2 levels, the method used was a cumulative logistic regression model. A power analysis (PROC POWER procedure in SAS 9.4) was used to compare the proportions of responses; a power greater than 0.8 is needed to conclude that people choose one choice significantly over another. Because each question was analyzed individually to produce a certain outcome, the participant data were analyzed separately.

Specifically, chi-square tests of independence were used to determine: (1) how many participants answered each question correctly compared to incorrectly for the questions that assessed consumers’ awareness of the impacts of different structural packaging designs (format + material) and packaging technologies on food freshness, with the null hypothesis that respondents chose both the right and the wrong answer equally, (2) whether the proportion of people choosing “Always” was larger than the proportion of “Sometimes” or “Never,” with the null hypothesis being that respondents chose evenly among the three options for the question that determines consumers’ purchase intent of packaging technologies after being educated, and (3) whether the proportion of people choosing “Yes” was larger than the proportion of “No” or “maybe,” with the null hypothesis being that respondents chose evenly among the three options for the question to determine consumers’ willingness to pay extra. Binary logistic regression was utilized to find significant differences along with two-way interactions with demographic and psychographic data in the case of the first two questions, while cumulative logistic regression was used to find the same information but for the question about willingness to pay. Furthermore, the chi-square independence test was used to determine whether there were any correlations between participants choices and each demographic/psychographic factor in each of the questions. Furthermore, a power analysis was carried out to compare the percentages of yes to no and yes to maybe.

## 3. Results and Discussion

### 3.1. Demographics and Psychographics

The demographic breakdown of the survey participants (Table 5) shows that the dominant groups were Caucasians, non-disabled, and non-Hispanic, Latino, or Spanish participants. Comparing this breakdown with the demographic breakdowns of the studies in Table 1, our study collected the behavior of US consumers who were the same as those in studies [25,30,31,32,33]. The rest of the studies in Table 1 were mainly performed in Europe. Regarding gender, our study presents a surveyed population almost evenly distributed between males and females. A similar split for gender can be found in [10,24,29,31], while the participants in the rest of the studies in Table 1 were characterized by a higher share of female participants. In terms of educational background, most of the participants in our study had some college experience or had graduated with a degree (such as an associate’s, bachelor’s, or graduate degree), which was comparable to research conducted by [25,31,32]. The primary income category of the participants in our study was over USD 100,000, which was higher compared to the studies in Table 1. However, the number of participants with annual earnings between USD 20,000 and USD 75,000 were comparable to [22,32]. The psychographic breakdown of survey participants is also presented in Table 5. Most participants bought their food at physical stores, and more than half of them frequently did so once a week. Approximately 90% of the participants helped cut down on home waste and did so because either they do not believe in wasting food or they spent money on it.

### 3.2. Consumers’ Awareness of the Impact of Structural Packaging Design on Food Freshness

#### 3.2.1. Consumers’ Awareness of the Impact of Structural Packaging Design on the Freshness of Cherries

**Figure 1:**
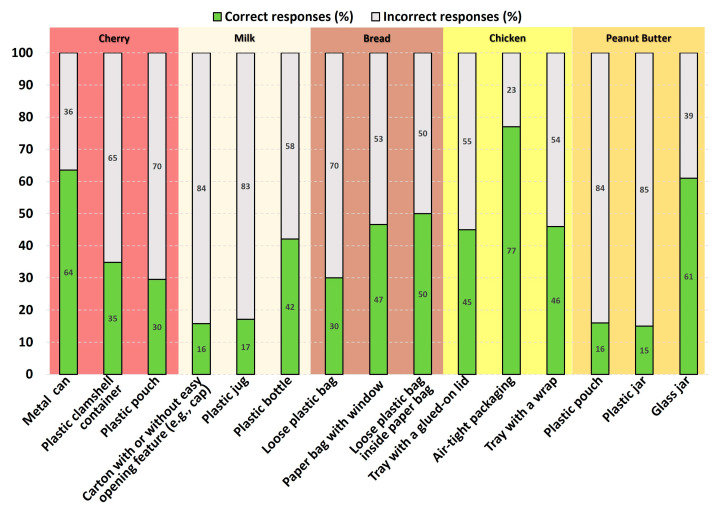
Consumers’ awareness (green) of the impact of structural packaging designs commonly found in US supermarkets (bottom) on maintaining the freshness of cherries, milk, bread, chicken, and peanut butter.

Figure 1 shows consumers’ awareness of the role different structural packaging designs commonly found in US supermarkets (Table 2) play in extending cherry shelf-life. Canning is commonly used to preserve fruits and vegetables for extended periods of time [9]. Plastic clamshell containers offer protection, air flow to prevent condensation and anaerobiosis, and stackability. Lately, plastic stand-up pouches have grown in use because of the usage of less plastic compared to the clamshell, thus aiming for sustainability. However, they provide less protection and allow for less air to flow. Approximately 64% of participants correctly selected the metal can as the structural package design that can keep cherries the freshest (*p* < 0.0001). Only ~35% and 30% of participants were able to identify the plastic clamshell container as neither the best nor the worst to extend cherry shelf-life (*p* < 0.0001) and the plastic pouch as the packaging design that keeps cherries fresh the least (*p* < 0.0001), respectively. The above shows that American consumers’ assessment of structural packaging designs for cherries is not quite correct. This most likely applies to other fresh produce with similar packaging and hence significantly contributes to household produce waste. These results could be explained by the significant number of American consumers who do not care about produce packaging [33]. Comparing our results with Langley et al.’ results [16], more Australian consumers feel that plastic packaging does not extend food shelf-life than American consumers.

Significant statistical differences among population segments were found only for educational background and ethnicity in the case of cherries in the metal can. Specifically, participants with a high school diploma, some college, an associate’s degree, and a bachelor’s degree indicated that the metal can will keep cherries freshest significantly more than participants with a different educational background (*p* < 0.0317). Participants who were not Hispanic, Latino, or of Spanish origin indicated that the metal can will keep cherries freshest significantly more than those who were of Hispanic, Latino, or Spanish origin (*p* < 0.0500).

#### 3.2.2. Consumers’ Awareness of the Impact of Structural Packaging Design on Milk Freshness

Figure 1 shows consumers’ awareness of the role different structural packaging designs play in extending the shelf-life of milk. In the US, high-temperature short-time (HTST) milk is sold in plastic jugs, whereas extended-shelf-life (ESL) or ultra-pasteurized (UHT) milk is commercialized in cartons and plastic bottles with opaque plastic sleeves (Table 2). These milks differ in shelf-life and, consequently, in packaging requirements. Under 20% of participants correctly identified the carton as the most (*p* < 0.0001) and the plastic jug as the least (*p* < 0.0001) effective structural package design for milk preservation. About 45% of participants correctly selected plastic bottles as neither the best nor the worst packaging for milk shelf-life extension (*p* < 0.0001). The above indicates that consumers misjudge milk structural package designs, which can greatly increase household milk waste. Additionally, Americans do not seem to read the label on milk packages, which discloses the processing conditions the milk underwent, or, if they do, they do not understand the meaning of the terms related to milk shelf-life. Hence, educating consumers to read labels or to learn about packaging technologies could contribute to reducing household milk waste. Contrary to Americans, South Africans were able to associate the carton box with long-term food storage [15]. The correct responses for the effect of the carton and the plastic jug on milk shelf-life were significantly different based on population segments. For both the carton and the plastic jug, female participants responded significantly better than male participants in selecting the carton as the best packaging design for maintaining milk’s freshness and the plastic jug as the worst packaging design for doing so (*p* < 0.0169 and *p* < 0.0167, respectively). Additionally, Black or African American, American Indian or Alaska Native, and Native Hawaiian or Other Pacific Islander participants selected the plastic jug as the worst packaging design in maintaining milk’s freshness significantly more than participants of a different race (*p* < 0.0041).

#### 3.2.3. Consumers’ Awareness of the Impact of Structural Packaging Design on Bread Freshness

Figure 1 shows consumers’ awareness of the role different structural packaging designs play in extending bread shelf-life. US retailers sell bread in a loose plastic bag made of a polyolefin to minimize staling, or in a paper bag for some basic protection if fast selling is expected, or in a combination of plastic and paper bags to increase bread shelf-life when needed (Table 2). Half of the participants correctly selected the loose plastic bag inside the paper bag as the package design that can keep bread the freshest (*p* = 1) and the paper bag as the least effective packaging design for bread freshness (*p* < 0.0315). Only 30% of participants identified the loose plastic bag as neither the best nor the worst packaging to extend bread shelf-life (*p* < 0.0001). The above shows that consumers’ assessment of packaging designs for bread is not quite correct, but much better than for cherries and milk. These findings also indicate that approximately half of the participants knew that a paper bag extends bread shelf-life less than a plastic bag. The correct responses for bread packaged in a paper bag and bread packaged in loose plastic bag inside the paper bag were significantly different based on population segments. Regarding bread packaged in a paper bag, participants with some school, some college, and a bachelor’s degree, those who were not of Hispanic, Latino, and/or Spanish origin, and those with incomes of at least USD 50,000 selected the paper bag as the packaging design that keeps bread fresh the least significantly more than participants from other groups within each respective population segment (*p* < 0.0339, *p* < 0.0005, and *p* < 0.0116). Regarding bread packaged in a loose plastic bag inside the paper bag, participants who shop for groceries once a week or more selected the loose plastic bag inside the paper bag as neither the best nor worst for maintaining bread freshness significantly more than participants who grocery shop every other week (*p* < 0.0113).

#### 3.2.4. Consumers’ Awareness of the Impact of Structural Packaging Design on Chicken Freshness

Figure 1 shows consumers’ awareness of the role different structural packaging designs commonly found in US supermarkets (Table 2) play in extending chicken shelf-life. These packages differ in headspace gas composition that impacts the oxidation and growth of anaerobic microorganisms differently, and, consequently, they have different barrier requirements. Many participants (77%) correctly identified air-tight packing as the most effective packaging design for maintaining chicken freshness (*p* < 0.0001). In total, 45 and 46% of participants rated the tray with a glued-on top as neither the best nor the worst packaging (*p* < 0.019) and the tray with a wrap as the least effective packaging for preserving chicken freshness (*p* < 0.0228), respectively. Consumers’ awareness about structural packaging designs for chicken was better than for cherries, milk, and bread. This higher awareness could be due to the advertisements of devices that remove air from pouches containing food to extend food shelf-life on television and through other media. The correct response given to each packaging design was significantly different based on population segments. Participants over the age of 41, participants who are not Hispanic, Latino, or Spanish, married, divorced, and widowed participants, participants who buy items at a physical store, and participants who contribute to reducing household food waste all selected air-tight packaging as the most effective design for maintaining chicken freshness significantly more than participants from other groups within each respective population segment (*p* < 0.0006, *p* < 0.0001, *p* < 0.0160, and *p* < 0.0240, and *p* < 0.0014). Furthermore, Asian, American Indian, or Alaska Native participants, participants who are not Hispanic, Latino, or Spanish, and those who grocery shop at least once per week all selected the tray with a wrap as the least effective design for maintaining chicken freshness significantly more than participants from other groups within each respective population segment (*p* < 0.0009, *p* < 0.0238, and *p* < 0.0006). American Indian or Alaska Native and Asian participants selected the plastic tray with a glued-on top as neither the best nor the worst packaging for maintaining chicken freshness significantly more than participants of a different race (*p* < 0.0308).

#### 3.2.5. Consumers’ Awareness of the Impact of Structural Packaging Design on Peanut Butter Freshness

Figure 1 shows consumers’ awareness of the role different structural packaging designs commonly found in US supermarkets (Table 2) play in extending peanut butter shelf-life. Glass jars, in contrast to plastic jars, are used to commercialize food for extended periods of time because glass is chemically inert, odorless, and impermeable to gases and vapors [7]. Stand-up pouches with dispensing devices have grown in popularity due to convenience. The presence of either metal foil or a metallized film in the pouch structure increases the odor, moisture, oil, water, and oxygen barrier, thereby extending food shelf-life longer compared to the plastic jar. Over half of participants (61%) correctly identified the glass jar to be the most efficient packaging for preserving peanut butter freshness (*p* < 0.0001). Venter et al. [15] found that South Africans believe that certain food products stored in glass packaging had a long shelf-life due to the high cost of glass. Less than 16% of participants correctly identified the pouch as neither the best nor the worst packaging (*p* < 0.0001), while 17% of them correctly selected the plastic jar as the least effective packaging design for maintaining peanut butter freshness (*p* < 0.0001). The information above demonstrates that consumers’ perceptions of peanut butter packaging designs in terms of freshness maintenance are not entirely accurate. The similar number of correct responses for both the plastic bag and the plastic jar could be attributed to participants’ perception of plastic without taking into consideration the aluminum foil or metallized film within the pouch, which enhances its barrier qualities, thereby helping prolong the peanut butter shelf-life in comparison to the plastic jar. The correct response for each of these three structural packaging designs was significantly different based on population segments. Participants aged 25 and under and 58 and over, and Caucasian, American Indian, or Alaska Native and Asian participants selected the glass jar as the best packaging design for maintaining the freshness of peanut butter significantly more than participants from other groups within each respective population segment (*p* < 0.0026 and *p* < 0.0053). Furthermore, participants with some college, an associate’s degree, a bachelor’s degree, Hispanic, Latino, or Spanish participants, participants with an income above USD 50,000, and participants who grocery shop more than once per week selected the plastic jar as the least effective packaging design for maintaining peanut butter freshness significantly more than participants from other groups within each respective population segment (*p* < 0.0198, *p* < 0.0480, *p* < 0.0044, and *p* < 0.0303). Participants with an income of >USD 20,000 and USD 50,000–USD 99,999 and those who shopped for groceries more than once a week and every other week rated the plastic pouch as neither the best nor the worst design for maintaining peanut butter freshness significantly more than participants from other groups within each respective population segment (*p* < 0.0500, *p* < 0.0101).

### 3.3. Consumers’ Awareness of the Impact of Packaging Technologies on Food Freshness

**Figure 2:**
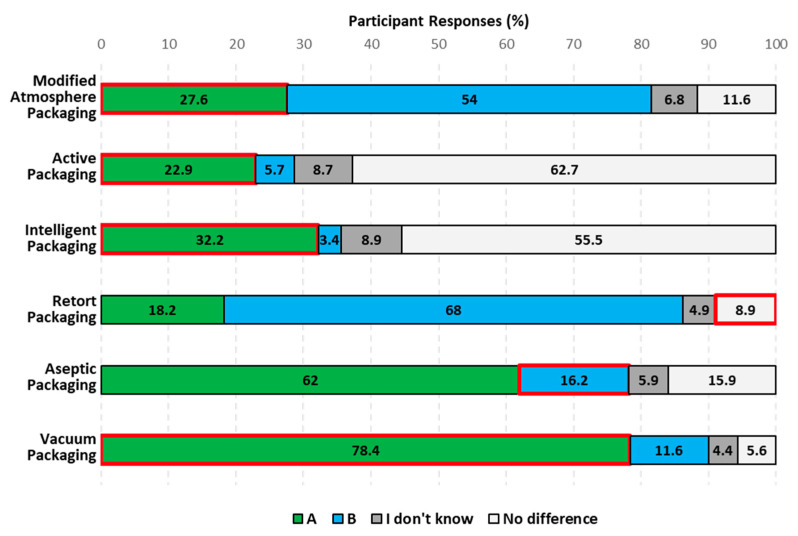
Consumers’ awareness of the ability of different packaging technologies to maintain food freshness or to provide information about the food product. Participant responses are presented in different colors: A (green), B (blue), I don’t know (dark gray), and no difference (light gray). A and B correspond to the left and right packages of each photo in Table 3. The correct response for each packaging technology is highlighted using a red rectangle.

Currently, the same food product can be found at the marketplace in packages that differ in their ability to extend food shelf-life or to provide information about the food product. MAP, AP, RP, ASP, and VP are all packaging technologies used to maintain food freshness. In contrast, IP is a packaging technology that provides information about the food product. Figure 2 presents consumers’ awareness of the ability of these packaging technologies to maintain food freshness longer.

#### 3.3.1. Consumers’ Awareness of the Impact of MAP on Food Freshness

MAP is a technology wherein the ambient air inside the package is replaced with a gas composition selected based on the needs of the food product for freshness maintenance and/or safety [17]. Participants were unaware of the effect that MAP plays on maintaining the freshness of food (specifically, fresh-cut lettuce) (Table 3), as shown by the significant number of incorrect responses (tray with a snap-fit lid, No difference, I don’t know) selected over the correct response (sealed pouch) (*p* < 0.0001). In fact, only 27.6% of participants selected the sealed pouch as the package that can keep the lettuce fresher for longer (Figure 2). US consumers’ lack of MAP recognition of 84% reported in the literature [30] may have contributed to the low number of correct responses. Consumers cannot be aware of the capacity of a technology to maintain food freshness if they are not familiar with such technology. Knowledge about MAP’s impact on maintaining food freshness was significantly different in relation to gender only, with male participants being more knowledgeable than female participants (*p* < 0.0001).

#### 3.3.2. Consumers’ Awareness of the Impact of AP on Food Freshness

AP is a technology wherein certain additives, referred to as “active compounds”, are added to the packaging material or placed inside the packaging container to improve food quality and/or safety [17]. Participants were unaware of the effect AP plays on extending the shelf-life of food products (specifically, beef jerky) (Table 3), as shown by the significant selection of the incorrect response (bag without a tiny packet, No difference, and I don’t know) over the correct response (bag with a tiny packet) (*p* < 0.0001). In fact, only 23% of participants selected the bag with a tiny packet as the package that can keep the beef jerky fresher for longer (Figure 2). Consumers’ recognition of AP rather than consumers’ knowledge of the impact of AP on food freshness has been studied (Table 1). Barska and Wyrwa [24] found that 42% and 16% of Polish consumers recognized AP when asked about packaging with scavengers to remove harmful gases to extend product durability and emitters to restrict microorganism growth, respectively. Studies performed in Ireland, Latvia, and Poland [26,27,28] have also shown that European consumers do not generally recognize AP. This lack of recognition could also be happening in the US, and it could be the reason why only 23% of Americans chose the correct response. If consumers are not able to recognize a packaging technology, it seems unlikely that they can know about its effect on food shelf-life. The selection of the choice that the bag with a tiny packet keeps the beef jerky fresher for longer was significantly different based only on participants’ contribution to reducing household food waste. Participants who contribute to reducing household food waste were more aware of the effect AP plays on extending the shelf-life of food products than participants who do not contribute to reducing household food waste (*p* < 0.0019).

#### 3.3.3. Consumers’ Awareness of the Impact of IP on Food Freshness

IP is a technology that monitors changes in the internal and external surroundings of the packed food product and conveys that information to facilitate decision making [36]. Furthermore, an intelligent package can give general information about the product through a barcode (QR code) [17]. Participants were unaware that IP (specifically, QR code) can provide them with information about the food (e.g., origin, freshness), specifically with regard to fresh-cut fruit (Table 3). This is shown by the significant selection of the incorrect response (container without a QR code, No difference, and I don’t know) over the correct response (container with a QR code) (*p* < 0.0001). In fact, only 32% of participants selected the container with a QR code as the one that can provide them with information (Figure 2). European consumers have little recognition of IP based on the literature. O’Callaghan and Kerry [27] found that 71.6% of Irish participants have never heard of IP. Similarly, Barska and Wyrwa [24] found that only 17% of Polish participants knew about IP. This lack of knowledge could be the same for Americans and the reason why they have no knowledge about a QR code providing information about the fresh-cut fruit. The awareness of IP information regarding food freshness was significantly different based on participants’ race, buying platform, grocery frequency, and contribution to reducing household food waste. Specifically, Caucasian, American Indian, or Alaska Native participants, those who buy items online and pick them up or have them delivered, those who grocery shop every other week or more than once a week, and those who contribute to reducing household food waste were more aware that IP (specifically, QR code) can provide them with information about food than participants from other groups within each respective population segment (*p* < 0.0452, *p* < 0.0333, *p* < 0.0495, and *p* < 0.0347).

#### 3.3.4. Consumers’ Awareness of the Impact of RP on Food Freshness

In RP, food is sealed in a glass, plastic, or metal container and heated to 121 °C or above for at least 10 min to ensure sterility for commercial usage [19]. Participants were not aware that tuna retorted in plastic pouches and cans possesses the same shelf-life (Table 3), as shown by the significant selection of the incorrect response (tuna in a pouch, canned tuna, and I don’t know) over the correct response (No difference) (*p* < 0.0001). In fact, only 9% of participants knew that both tuna in a pouch and canned tuna possess the same shelf-life (Figure 2). Based on these results, participants did not know that tuna retorted in plastic pouches and cans possesses the same shelf-life. This shows that participants associated the long shelf-life of the tuna to a specific packaging design. Awareness of tuna having the same freshness in a can and a plastic pouch was significantly different based on participants’ disability status. Participants with a disability were more aware that the same shelf-life for tuna is possible in both the can and the plastic pouch than participants without a disability (*p* < 0.0453). The difference in knowledge may be due to the need of Americans with specific disabilities to learn about packaging alternatives to cans that offer easier opening.

#### 3.3.5. Consumers’ Awareness of the Impact of ASP on Food Freshness

ASP involves the packing of a sterilized food product into sterilized containers that are sealed in a commercially sterile environment to eliminate microorganisms [20]. Because of the low microbial load, ASP is commonly made of paper, aluminum, and plastic to maintain the quality of the food product without the need for refrigeration. Participants were unaware of the effect ASP plays in extending the shelf-life of food products (specifically, juice) (Table 3), as shown by the significant number of selected incorrect responses (plastic jug, No difference, I don’t know) over the correct response (carton) (*p* < 0.0001). In fact, only 16% of participants selected the carton as the package that can keep the orange juice longer (Figure 2). Thus, no differences within the same population segment were observed for ASP.

#### 3.3.6. Consumers’ Awareness of the Impact of VP on Food Freshness

VP is a technology that removes the air from the package headspace to eliminate oxygen, thus creating an anaerobic environment that prevents the growth of spoilage bacteria and decreases the pace of oxidative processes that cause product degradation to increase shelf-life [9]. It also increases food shelf-life by reducing freezer burn because of the absence of package headspace [9]. The impact of VP on maintaining food freshness (Table 3) was acknowledged throughout the participants, as shown by their significant selection of the air-tight package over the incorrect response (non-air-tight package, no different, I don’t know) (*p* < 0.0001) for the packaging that can keep salmon fresher for longer. In fact, most participants (78%) chose the air-tight package (Figure 2). Likewise, Bou-Mitri et al. [10] reported that most Lebanese consumers thought that vacuum-packaged cheese had the greatest quality when asked to compare different types of packaging for cheese. In addition, Chen et al. [22] reported that 76% of Canadian consumers said they had heard of vacuum packaging and understood its function. Americans selected the air-tight package differently based on their age, gender, race, marital status, and educational background. Participants who were 42+ years old, female participants, Caucasian and American Indian/Alaska Native participants, married, separated, divorced, and widowed participants, and those who had an educational background that included a high school diploma, some college experience, and an associate’s degree were more aware of VP’s impact on maintaining food freshness than participants from other groups within each respective population segment (*p* < 0.0001, *p* < 0.0020, *p* < 0.0001, *p* < 0.00010, and *p* < 0.0419).

### 3.4. Assessing Consumers’ Purchase Intent towards Packaging Technologies after Being Educated

**Figure 3:**
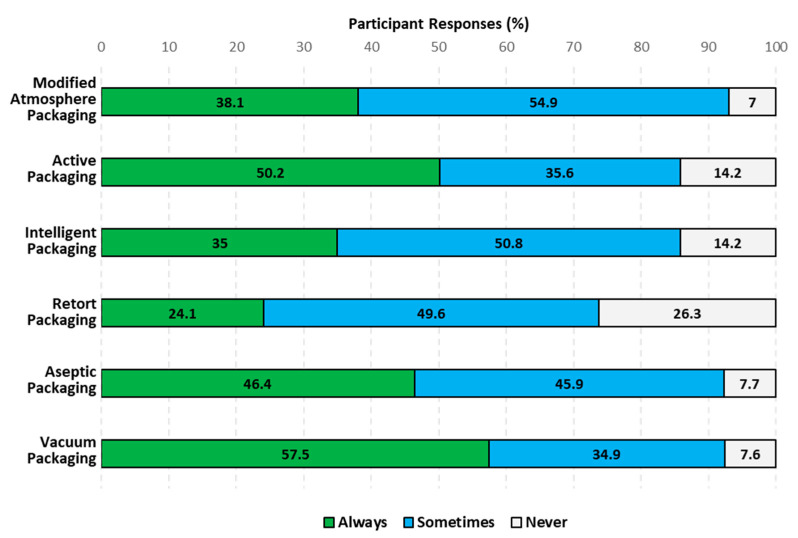
Consumers’ purchase intent of food commercialized in MAP, AP, IP, RP, ASP, and VP after learning about these packaging technologies.

Figure 3 presents participants’ purchase intent of food commercialized in packaging technologies that can contribute to reducing food waste (MAP, AP, IP, RP, ASP, and VP) after being shown the same images as in Section 3.3 (Table 3) along with the corresponding definition of each of the technologies (Table 4).

#### 3.4.1. Consumers’ Purchase Intent of MAP

Participants were willing to purchase food products in MAP always and sometimes more than never (*p* < 0.0001). The “always” and “sometimes” responses made up a total of 93% participants willing to buy food products in the packaging technology (Figure 3). However, Americans showed significant differences in the purchase intent of food products in MAP within the same population segment. Younger American consumers (>41 years old), those who have never been married, those who are divorced, and those who go grocery shopping one or more times per week reported they would purchase MAP always and sometimes significantly more than American consumers from other groups within each specific population segment (*p* < 0.04, *p* < 0.02, and *p* < 0.01). Two-way interactions between population segments were not found for MAP. Acceptance rather than purchase intent of MAP has been studied in the past. Specifically, 55% of European consumers accepted MAP as a technology to improve cheese safety [23].

#### 3.4.2. Consumers’ Purchase Intent of AP

Participants were willing to purchase food products in AP always and sometimes more than never (*p* < 0.0001). The “always” and “sometimes” responses made up a total of 86% participants open to purchasing food products in the technology (Figure 3). Comparing the purchase intent of AP by Americans with other nationalities (Table 1), a lower inclination to buy AP has been reported for Europeans. Barska and Wyrwa [24] found that 68% of Polish participants were willing to purchase food in AP after receiving a brief explanation of the packaging technology, and O’Callaghan and Kerry [27] reported a much lower purchase intent of cheese (10%) in AP by Irish participants after also being educated. Americans showed significant differences in the purchase intent of food products in AP within the same population segment. Americans between the ages of 18 and 57 responded that they would purchase AP always and sometimes significantly more than participants above the age of 57 (*p* < 0.0001). Likewise, O’Callaghan and Kerry [27] found that older Irish participants were more inclined to desire no technological interference. American males responded that they would purchase AP always and sometimes significantly more than American females (*p* < 0.0008). This could be attributed to American males liking AP more than American females [33]. Caucasian and Black or African American participants and participants who grocery shop more than once per week also responded that they would purchase AP always and sometimes significantly more than participants of a different group within the same population segment (*p* < 0.0049 and *p* < 0.0328). Several two-way interactions between population segments were identified (*p* < 0.05), and the details can be found in the Supplementary Information (Supplementary S1).

#### 3.4.3. Consumers’ Purchase Intent of IP

Participants were willing to purchase food products in IP always and sometimes more than never (*p* < 0.0001). The “always” and “sometimes” responses made up a total of 86% participants open to purchasing food products in the technology (Figure 3). Previous research conducted in other countries has shown positive IP buying intentions, thus supporting our findings. Baska and Wyrwa [24] found that 67% of Polish consumers were open to purchasing IP after learning about it. O’Callaghan and Kerry [27] and Cammarelle, Viscecchia, and Bimbo [34] reported that Irish and Italian consumers, respectively, were more open to purchasing IP than AP after getting information about the technology. Our findings indicate the opposite for US consumers, who were more willing to buy AP than IP. Americans showed significant differences in the purchase intent of food products in IP within the same population segment. All races except for Caucasians, Asians, and Hispanic, Latino, or Spanish participants would purchase IP always and sometimes significantly more than participants of a different group within the same population segment (*p* < 0.0052 and *p* < 0.0306). Several two-way interactions between population segments were identified (*p* < 0.05). These can be found in the Supplementary Information (Supplementary S1).

AP and IP were the two technologies with the highest number of “never” responses after RP. This finding is supported by several studies that highlight that consumers’ lack of trust/skepticism towards new packaging technologies is another issue impeding their functionality in food saving [24,29].

#### 3.4.4. Consumers’ Purchase Intent of RP

Participants were willing to purchase tuna or another food product in a pouch instead of in a can sometimes more than either always or never (*p* < 0.0001). The “sometimes” responses made up half of the participants (Figure 3). A lower percentage of “always” and a higher percentage of “never” compared to other packaging technologies was obtained for RP. This finding may indicate that US consumers have a strong attachment to certain packaging designs and are unwilling to changes them. Americans showed significant differences in the purchase intent of food products in RP within the same population segment. Participants under the age of 58 would purchase food in retort pouches always and sometimes significantly more than participants 58 and older (*p* < 0.0002). This could be justified by the growth in difficulty of opening packages with age. Furthermore, participants who contribute to reducing household food waste also responded that they would purchase food in retort pouches always and sometimes significantly more compared to participants who do not contribute to reducing household food waste (*p* < 0.0109). The details of each of the numerous two-way interactions that were identified between population segments can be found in the Supplementary Information (Supplementary S1).

#### 3.4.5. Consumers’ Purchase Intent of ASP

Participants were willing to purchase food products in ASP always and sometimes more than never (*p* < 0.0001). The “always” and “sometimes” responses made up a total of 92% participants willing to buy food products in the technology (Figure 3). Considering that just a small portion (16%) of the participants were aware of the technology’s ability to extend food shelf-life (Figure 2), this is a considerable change that indicates how education can help with the purchase of technologies that can reduce food waste. Americans showed significant differences in the purchase intent of food in ASP within the same population segment. Caucasian and Black or African American participants and those who buy items online and have them delivered would purchase ASP always and sometimes significantly more than participants of a different group within the same population segment (*p* < 0.0196 and *p* < 0.0472). Furthermore, one two-way interaction between age and income was identified, and it can be found in the Supplementary Information (Supplementary S1).

#### 3.4.6. Consumers’ Purchase Intent of VP

Participants were willing to purchase food products in VP always and sometimes more than never (*p* < 0.0001). The “always” and “sometimes” responses made up a total of 93% participants willing to buy food products in the technology (Figure 3). The reason for this high number must have been the participants’ awareness of the positive impact of the technology on maintaining food freshness, which is already shown in Section 3.2. However, the number of participants who declared an intention to purchase VP was higher (93%) (Figure 3) than those who recognized VP as a technology able to extend shelf-life (78%) (Figure 2), which demonstrates the impact of consumer education. Participants showed significant differences in the purchase intent of food products in VP within the same population segment. Participants with some college experience or a bachelor’s degree would purchase food in VP always and sometimes significantly more than participants with a different educational background (*p* < 0.0398). Likewise, Chen, Anders, and An [22] found that consumers with a reasonably high level of education were more supportive of VP because they were already aware of and familiar with it. Non-disabled participants would buy food in VP always and sometimes significantly more compared to participants with a disability (*p* < 0.03). This could be because of the difficulty of opening VP compared to a tray wrapped with a film. Furthermore, participants who contribute to reducing household food waste would buy food in VP always and sometimes significantly more compared to participants who do not contribute to reducing household food waste (*p* < 0.0233). Two-way interactions were not found for vacuum packaging. Table 1 shows that the prior literature on VP [22,23,32] does not include consumers’ purchase intentions but simply their acceptability and willingness to pay for VP’s capacity to increase food shelf-life. For example, Chen, Anders, and An [22] studied Canadian consumers’ acceptability of vacuum-packaged beef steak.

### 3.5. Willingness to Pay Extra for Food Products in Packages Designed to Help Reduce Food Waste

Price influences both food purchase decisions and food waste, according to the literature [27,37,38]. Several studies have been conducted to determine if consumers are willing to pay extra for packaging technologies that extend food shelf-life (Table 1), but nothing is known about consumers’ willingness to pay extra for food in packages designed to help reduce food waste. This study shows that there was a willingness to pay (*p* < 0.0001). Specifically, 47% and 38% of participants responded “yes” and “maybe”, respectively, when asked for their willingness. Furthermore, “Yes” was chosen significantly more often than “maybe” (*p* < 0.05). This high number of US consumers willing to pay extra for food in packages designed to help reduce food waste aligns with the reported consumers’ willingness to pay to reduce food waste [39] and to pay extra for packaging technologies intended to increase food shelf-life [10,24,30,31,33]. The expected higher price of food packaged in novel packaging technologies [24] may explain the 15% of “No” replies in this study.

This willingness to pay extra was different among participants. Participants with a disability and those who contribute to reducing household food waste were more willing to pay extra for food products in packages designed to help reduce food waste compared to participants of a different group within the same population segment (*p* < 0.05 and *p* < 0.0001). Furthermore, the willingness to pay extra was different between population segments, as shown by the several two-way interactions found in this study. The details can be found in the Supplementary Information (Supplementary S2).

## 4. Conclusions

This study investigates the knowledge that consumers have about the impact of packaging on food freshness for the first time. US consumers had little knowledge about the impact of both structural packaging designs and packaging technologies on food freshness, which prevents them from making the correct shopping choices to maintain food freshness and thereby reduce household food waste. However, upon learning what these packaging technologies entail, most of the US consumers would always/sometimes buy food in packaging that can contribute to reducing food waste, with flexible RP being the lone exception. Therefore, education can influence US consumers’ opinions on packaging technologies, which can significantly cut the amount of food waste. VP, AP, and ASP are the three packaging technologies that US consumers are most likely to purchase food products in; thus, these could be the target packaging technologies to reduce American household food waste. Contrary to the results of other survey studies conducted outside of America, the probability of US consumers purchasing food in AP is higher than the probability of purchasing food in IP. In addition to the high purchase intent of packaging designed to reduce food waste by US consumers, most of them are willing to pay extra for food commercialized in it. US consumers’ attitudes towards awareness, purchase intent, and willingness to pay extra varied across demographic characteristics, which shows that targeting specific population segments can make a big difference when implementing strategies to reduce household food waste. While differences within the same population segments were often found for race, grocery frequency, and participants’ contribution to food waste reduction, these were never found for household size. All in all, this study has generated a new and deeper understanding about the intersections between packaging and American household food waste that can contribute to designing new packaging, developing effective education programs, and making new policies. The implementation of the above can greatly aid consumers’ capability to make the proper choices when selecting packaging technologies and designs that maintain food freshness during shopping, thereby contributing to reducing household food waste.

## Figures and Tables

**Table 2:** Packages for cherries, milk, bread, chicken, and peanut butter presented to the survey participants.

Food Product	Structural Packaging Designs		
Cherries	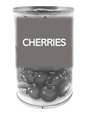 Metal can	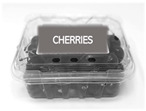 Plastic clamshell container	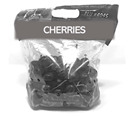 Plastic pouch
Milk	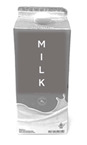 Carton	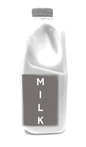 Plastic jug	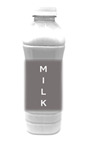 Plastic bottle
Bread	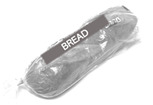 Loose plastic bag	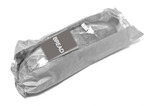 Paper bag with window	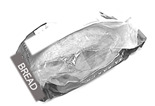 Loose plastic bag inside paper bag
Chicken	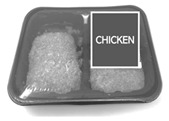 Plastic tray with a glued-on lid	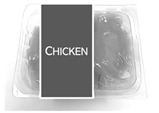 Plastic air-tight packaging	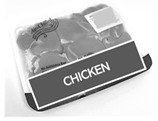 Plastic tray with a wrap
Peanut butter	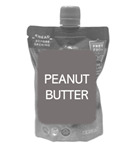 Plastic pouch	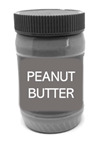 Plastic jar	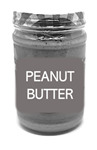 Glass jar

**Table 3:** Packaging technologies including MAP, AP, IP, RP, ASP, and VP presented to the survey panelists.

Packaging Technology	Left (A); Right (B)	Packaging Technology	Left (A); Right (B)
MAP	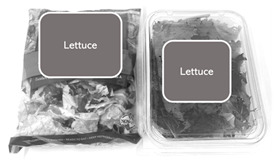	**RP**	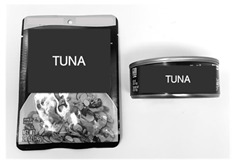
AP	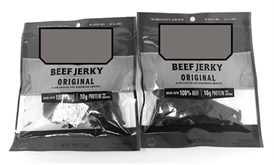	**ASP**	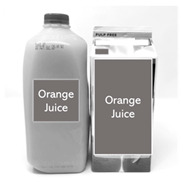
IP	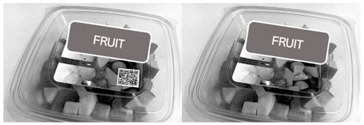	**VP**	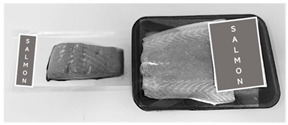

**Table 4:** Text provided for MAP, AP, IP, RP, ASP, and VP to the participants.

Packaging Technology	Definition
MAP	Package A is a modified atmosphere package, which means that package A is a hermetically sealed package that contains a safe gas that keeps the fresh-cut lettuce fresher. Package B is not hermetically sealed, which means the package contains air, which spoils the fresh-cut lettuce faster.
AP	Package A is an active package, which means that package A contains an insert (little packet that removes oxygen) to keep the beef jerky fresher. Package B does not contain this insert, so the product will spoil faster.
IP	Package A is an intelligent package because it has an artifact (QR code) that provides you with information on the fresh-cut fruit (e.g., origin, freshness). Package B does not have an artifact to provide this information.
RP	Packages A and B are both retort packages, which means that the tuna is exposed to a high temperature while inside the packages to kill any microorganisms present in order to maintain freshness.
ASP	Package B is an aseptic package, which means the orange juice has been packaged in an environment free of microorganisms to be kept fresher. Package A has not undergone the aseptic process.
VP	Package A is a vacuum package, which means the air has been eliminated from the package before sealing to keep the salmon fresher. Package B is not vacuum packaged, which means it contains air that will spoil the salmon faster.

**Table 5:** Participants’ demographic and psychographic breakdowns.

Population Segments	Population Groups	Participants (%) n = 1000
**Demographics**		
Gender	Male	46.9
	Female	53.1
Age	18–25	6.3
	26–41	27.8
	42–57	28.5
	58+	37.4
Race	White	76.5
	Black or African American	13.2
	American Indian or Alaska Native	3.8
	Asian	5.6
	Native Hawaiian or Other Pacific Islander	0.9
Income	>USD 20,000	13.0
	USD 20,000–USD 49,999	27.9
	USD 50,000–USD 74,999	19.9
	USD 75,000–USD 99,999	10.3
	<USD 100,000	28.9
Educational Background	Some school	2.1
	High school diploma or GED	22.1
	Some college	28
	Associate’s degree or 2-year degree	11.9
	Bachelor’s degree or 4-year degree	21.9
	Graduate degree or more	14.0
Marital Status	Married	45.6
	Never married	34.1
	Separated	2.1
	Divorced	11.8
	Widowed	6.4
Household Size	1 person	26.1
	2 persons	35.9
	3 persons	15.6
	4 persons	13.3
	5 persons	6.2
	6 persons and up	2.9
Disability	Yes	15.2
	No	84.8
Ethnicity	Hispanic, Latino, or Spanish origin	18.5
	Not Hispanic, Latino, or Spanish origin	81.5
Region	Northeast	17.9
	Midwest	21.8
	West	22.2
	South	38.1
**Psychographics**		
Grocery Method	Buy items online and then go and pick them up	6.9
	Buy items online and have them delivered to you	12.1
	Buy at a physical store	81.0
Grocery Frequency	Every other week	22.4
	Once per week	52.5
	More than once per week	25.1
Reduce Waste	Yes	90.3
	No	9.7
Why do you try to throw away less food?	I spent money on that food	33.1
	I do not believe in wasting food	56.9
	I do not want it to negatively impact the environment	9.4
	I am not sure	0.5

## Data Availability

The data used to support the findings in this study may be made available by the corresponding author upon request.

## Supplementary Materials

The following supporting information can be downloaded at: https://www.mdpi.com/article/10.3390/foods12234315/s1, Supplementary S1: Consumers’ purchase intent of packaging technologies that can reduce food waste: two-way interactions; Supplementary S2: Willingness to pay extra for food in packaging intended to reduce food waste: two-way interactions. Refs. [40,41,42,43] are cited in Supplementary Materials.
