# Supplementary material for: US Consumers’ Awareness, Purchase Intent, and Willingness to Pay for Packaging That Reduces Household Food Waste
Source: Foods. 2023 Nov 29;12(23):4315. doi: 10.3390/foods12234315 (PMC10705878; doi:10.3390/foods12234315)
Supplement: Supplementary file 1 [file foods-12-04315-s001.zip › foods-2717230-supplementary/S1-Fennell et al-Waste#2-.pdf]

S1. Consumers' purchase intent of packaging technologies that can reduce food waste: 2-way interactions

Consumers' purchase intent of some of the packaging technologies that can reduce food waste (AP, IP, report packaging, and aseptic packaging) differed among population segments as shown by the several 2-way interactions found in this study, which are discussed below.

AP: Two-way interactions between gender and education and income were found. Male participants with some college experience responded they would purchase AP always and sometimes less than male participants with a different educational background. Male participants who earn between $75,000 and $99,999 responded they would purchase AP always and sometimes more than males with a different income level.

Two-way interactions between age and grocery shopping frequency and income were found. Millennials (26 to 41 years old) who grocery shop every other week or once per week responded they would purchase AP always and sometimes less than millennials who grocery shop more than once per week. Likewise, Gen X (42 to 57 years old) participants that grocery shop every other week or more than once per week responded they would purchase AP always and sometimes less than gen Z participants who grocery shop once per week. Millennials (26 to 41 years old) participants who earn between $75,000 and $99,999 responded they would purchase AP always and sometimes more than millennials with a different income level.

One two-way interaction between ethnicity and income was found. Hispanic, Latino, or Spanish who earn between $50,000 and $99,999 responded they would purchase AP always and sometimes less than participants of this ethnic group with a different income.

Two two-way interactions between education and grocery shopping frequency and reduce waste were found. Participants with a high school diploma, associate's degree, and/or bachelor's degree who grocery shop every other week responded they would purchase AP always and sometimes less than participants with the same educational background but a different grocery shopping frequency.

Three two-way interactions between income and grocery shopping frequency were found. Participants who earn between $50,000 and $74,999 while grocery shop every other week responded they would purchase AP always and sometimes less than participants with the same income with a different grocery shopping frequency. Likewise, participants who earn between $75,000 and $99,999 and grocery shop every other week or once per week responded they would purchase AP always and sometimes less than participants with the same income with a different grocery shopping frequency.

Two-way interaction between contribution to reducing household food waste and education and grocery shopping frequency were found. Participants with some college experience who contribute to reducing household food waste responded they would purchase AP always and sometimes less than participants with the same educational background who do not contribute to reducing household food waste. Likewise, participants who grocery shop once per week while contributing to reducing household food waste responded they would purchase AP always and sometimes less than participants with the same grocery shopping frequency who do not contribute to reducing household food waste.

IP: Two two-way interactions between age and gender and education were found. Male Gen X (42 to 57) participants responded they would purchase IP always and sometimes more than female Gen Z participants. Furthermore, Gen X participants with some college experience responded they would purchase IP always and sometimes more than Gen Z participants with a different educational background.

Two two-way interactions between education and income and grocery shopping frequency were found. Participants with some college experience who earn between $50,000 and $74,999 annually responded they would purchase IP always and sometimes less than participants with the same educational background with a different income. In contrast, participants with a high school diploma or some college experience who grocery shop once per week responded they would purchase IP always and

sometimes more than with the same educational background with a different grocery shopping frequency. Participants who earn between $75,000 and $99,999 annually and grocery shop once per week responded they would purchase IP always and sometimes less than participants with the same income with a different grocery shopping frequency.

One two-way interaction between disability and grocery shopping frequency was found. Disable participants who grocery shop once per week responded they would purchase IP always and sometimes less than disable participants with a different grocery shopping frequency.

Retort packaging: One two-way interaction between education and race was found. White and Black or African American participants with a bachelor's degree responded they would purchase retort packaging always and sometimes less than participants who belong to the same races with a different educational background.

Two-way interactions between education and income, marital status, and contribution to reducing household food waste were found. Participants with a high school diploma and/or some college experience who earn between $20,000 and $49,999 & $75,000 and $99,999 responded they would purchase retort packaging always and sometimes less than participants with same educational but a different income. Similarly, participants with an associate’s degree who earn between $75,000 and $99,999 responded they would purchase retort packaging always and sometimes less than participants with same educational but a different income. Participants with high school diploma, some college experience, or a bachelor's degree who are divorced responded they would purchase retort packaging always and sometimes more than participants with the same educational background but different marital status. Participants with some college experience who contribute to reducing household food waste responded they would purchase retort packaging always and sometimes more than participants with the same educational background who do not contribute to reducing food waste.

One two-way interaction between ethnicity and income was found. Hispanic, Latino, or Spanish participants who earn between <$20,000 and between $50,000 and $99,999 responded they would purchase retort packaging always and sometimes more than participants of the same ethnic group with a different income.

One two-way interaction between disability and income was found. Disable participants who earn less than $20,000 responded they would purchase retort packaging always and sometimes less than disable participants with a different income.

Three two-way interactions between income and marital status, grocery shopping method, and contribution to reducing household food waste were found. Participants that earn between $20,000 and $49,999 and are divorced responded they would purchase retort packaging always and sometimes less than participants with the same income but a different marital status. Likewise, participants who earn this same income and contribute to reducing household food waste responded they would purchase retort packaging always and sometimes less than participants that do not contribute to reducing household food waste. Participants who earn between $50,000 and $74,999 and buy items online and have them delivered responded they would purchase retort packaging always and sometimes less than participants with the same income with a different grocery shopping method.

Two two-way interactions between gender and grocery shopping method and contribution to reducing household food waste were found. Male participants who buy items online and pick them up responded they would purchase retort packaging always and sometimes less than male participants with a different grocery shopping method. In contrast, male participants who contribute to reduce household food waste responded they would purchase retort packaging always and sometimes more than male participants that do not contribute to reducing household food waste.

One two-way interaction between grocery shopping method and grocery shopping frequency was found. Participants who buy items online and have them delivered while grocery shopping once per

week responded they would purchase retort packaging always and sometimes more than participants with the same grocery shopping method but a different grocery shopping frequency.

Several two-way interactions between age and education, ethnicity, income, grocery shopping method, and grocery shopping frequency were found. Millennial (26-41 years old) participants with a high school diploma, some college experience, and/or an associate's degree responded they would purchase retort packaging always and sometimes more than millennials with a different educational background. Gen Z (18-25 years old) participants who identify as Hispanic, Latino, or Spanish responded they would purchase retort packaging always and sometimes less than non- Hispanic, Latino, or Spanish Gen Z participants. Likewise, Gen Z participants who earn less than $20,000 responded they would purchase retort packaging always and sometimes less than Gen Z participants with a different income. Millennials (26-41 years old) who earn between less than $20,000 and $49,999 responded they would purchase retort packaging the always and sometimes less than millennials with a different income. Likewise, millennials who earn between $75,000 and $99,999 responded they would purchase retort packaging the always and sometimes less than millennials with a different income. Millennials who buy items online and have them delivered responded they would purchase retort packaging always and sometimes more than millennials with a different grocery shopping method. Gen X (42-57 years old) participants who buy items online and pick them up and/or buy items online and have them delivered responded they would purchase retort packaging always and sometimes more than Gen X participants who buy items at the physical store. Gen X participants who grocery shop every other week responded they would purchase retort packaging always and sometimes less than Gen X participants with a different grocery shopping frequency.

Aseptic packaging: One two-way interaction between age and income was found. Gen X (18-25 years old) participants who earn between $50,000 and $74,999 responded they would purchase aseptic packaging always and sometimes more than Gen X participants with a different income.
