# Supplementary material for: US Consumers’ Awareness, Purchase Intent, and Willingness to Pay for Packaging That Reduces Household Food Waste
Source: Foods. 2023 Nov 29;12(23):4315. doi: 10.3390/foods12234315 (PMC10705878; doi:10.3390/foods12234315)
Supplement: Supplementary file 1 [file foods-12-04315-s001.zip › foods-2717230-supplementary/S2-Fennell et al-Waste#2-.pdf]

S2. Willingness to pay extra for food in packaging intended to reduce food waste: 2-way interactions.

This willingness to pay extra differed among population segments as shown by the several 2-way interactions found in this study, which are discussed below.

The Gen Z participants (18-25 years old) who identify as Hispanic, Latino, or Spanish were more willing to pay extra compared to non- Hispanic, Latino, or Spanish Gen Z participants. In contrast, Gen X participants (42-57 years old) who identify as Hispanic, Latino, or Spanish were less willing to pay extra compared to non- Hispanic, Latino, or Spanish Gen X participants. Gen Z participants who contribute to reducing household food waste were more willing to pay extra compared to participants in the same age group who do not contribute to reducing household food waste. In contrast, Gen X participants who contribute to reducing household food waste were less willing to pay extra compared to Gen X participants who do not contribute to reducing food waste. The above results show how groups of generations X and Z differ from each other in the willingness to pay extra, which was not the case for other age generations. Wilson et al. [33] reported that participants under the age of 25 were willing to spend more money on packaging that can extend the usage of a food product while respondents over the age of 25 were not. Based on the above findings, two population segments with a similar age split for willingness to pay extra or not for food products in packaging intended to assist reduce food waste were identified: Hispanic, Latino, or Spanish, and participants who help reduce food waste.

Black or African American participants who earn <$20,000 were more willing to pay extra compared to participants of the same race with a different income. In the case of the Hispanic, Latino, or Spanish participants, the ones who earn between $20,000 and $49,999 were less willing to pay extra compared to Hispanic, Latino, or Spanish participants with a different income. Furthermore, Gen X participants who earn between $75,000 and $99,999 were less willing to pay extra compared to participants in the same age group with a different income. Therefore, the effect income on willingness to pay extra varies depending on population segments (group age, race, and ethnicity). According to multiple studies [40-41], higher-income households waste more food. Therefore, people with high annual incomes should not see value in spending more money on food items in packaging that can decrease food waste. However, our findings do not support such a relationship.

Black or African American participants who are married were less willing to pay extra compared to Black or African American participants who have never been married, separated, divorced, or widowed. In contrast. married participants who contribute to reducing household food waste were more willing to pay extra compared to married participants who do not contribute to reducing household food waste.

Disable participants who buy items online and pick them up were less willing to pay extra compared to disable participants with a different grocery shopping method. In contrast, Hispanic, Latino, or Spanish participants who buy items online and pick them up were more willing to pay extra compared to Hispanic, Latino, or Spanish participants who buy items online and have them delivered or buy items from a physical store. Male participants who buy items online and have them delivered were less willing to pay extra compared to male participants with a different grocery shopping method. Similarly, Gen X who buy items online and have them delivered were less willing to pay extra compared to Gen X participants with a different grocery shopping method. Based on the above results, the willingness to pay extra is different between population segments if the food purchase occurs online but not if this occurs at a physical store.

Participants with an associate's degree who grocery shop once per week were more willing to pay extra compared to participants with the same educational background who grocery shop every other week or more often. In contrast, Black or African American participants who grocery shop once per week were less willing to pay extra compared to Black or African American who grocery shop every other week or more often. Gen Z participants who grocery shop every other week were less willing to pay extra compared to participants of the same age group with a different grocery shopping frequency. Participants that grocery shop every other week or once per week who contribute to reducing household food waste

were willing to pay extra compared to participants with the same grocery shopping frequency that do not contribute to reducing household food waste. Previous household food waste studies have reported that families who shop more regularly typically waste less food [42-43]. In principle, families who shop less regularly should be more willing to pay extra. However, our findings show that such willingness varies depending on the population group.
